# Lipedema: clinical characteristics, complications, and the importance of evidence-based practice

**DOI:** 10.1590/1806-9282.20240801

**Published:** 2024-09-13

**Authors:** Ana Carolina Padilha de Paula, Jônatas de Oliveira

**Affiliations:** 1 Universidade Federal de São Paulo – São Paulo (SP), Brazil.; 2 Institute de Pesquisa em Comportamento e Comida – São Paulo (SP), Brazil.; 3 Universidade de Sao Paulo, Faculdade de Medicina – São Paulo (SP), Brazil.

## INTRODUCTION

Lipedema is a chronic condition involving the abnormal accumulation of fat, primarily in the legs, thighs, and, in some cases, the arms. It was recently included in the 11th edition of the International Classification of Diseases, which highlights the growing awareness of this disease^1-3^. Historically, it was first described by Allen and Hines^4^, but even today, it is underdiagnosed and poorly understood. The estimated prevalence in Brazil is 12.3%^5^. However, the lack of knowledge about lipedema often leads to incorrect diagnosis, with the condition being confused with other conditions such as obesity and lymphedema.

The most significant challenge currently facing the field is the dearth of evidence-based approaches and the heated debates, particularly in the context of rapidly evolving media such as social networks^6^. Additionally, the emergence of numerous specialists and a multitude of treatment options has further complicated matters. This article will examine the clinical characteristics of lipedema, its associated complications, the necessity of evidence-based practice, and the guidelines available for its treatment.

## CLINICAL DESCRIPTION

Lipedema is defined by a disproportionate distribution of body fat, bilateral and symmetrical enlargement of the limbs, and weight loss with limited or no significant influence. Patients frequently report pain, increased sensitivity to touch, easy bruising, and fatigue of the affected limbs^1-3^. Edema, when present, is minimal, and there is no reduction in pain with limb elevation. The disease does not affect the hands or feet. Patients often present with a clinical picture of "painful fat" due to sensitivity to touch and capillary fragility^7-10^.

The type of fat characteristic of the disease does not evolve in the same way as in the rest of the body. Further studies are necessary to elucidate the role of hormonal fluctuations, interferences, and drug interactions and their evolution in periods of major hormonal changes such as puberty, pregnancy, and menopause^11^. The disease has four types that take into account the location of the fat^12^, as described in Table 1.

**Table 1:** Presentations of lipedema.

Stages	Types
Stage I: The surface of the skin is normal, and the subcutaneous fatty tissue has a soft consistency. However, multiple small nodules can be palpated.	Type I: presents as an increase in fatty tissue on the buttocks and thighs.
Stage II: The surface of the skin becomes uneven and stiffer due to the increased formation of nodular structures (large lumps) in the subcutaneous fatty tissue, a process called liposclerosis.	Type II: involves the extension of edema and the formation of fatty deposits on the inside of the knees.

## COMPLICATIONS

In addition to physical pain and edema, lipedema can cause significant psychological discomfort. Negative body image and the potential development of maladaptive eating behaviors are critical concerns^6^. Reports suggest that women with lipedema may experience binge eating, food restriction, and compensatory practices^7-10^. Taking into account the late diagnosis, the reports are of tireless searches for treatments of the most diverse kinds, which have ended up increasing the sense of failure, impotence, and insufficiency of these women, who have also been subjected to professionals with less than empathetic discourses and surrounded by prejudices and little knowledge of the condition, which at all times invalidate the possible small results^11-13^.

## TREATMENT

Conventional weight-loss treatments are often ineffective for patients with lipedema due to the inflammation and fibrosis of the affected adipose tissue. Inadequate interventions, which do not take into account patients’ individuality and diet history, can result in frustration and feelings of guilt. Based on no robust ­evidence, there is a debate among doctors about the relative merits of conservative versus surgical treatment. Testimonies from women with lipedema demonstrate the adverse impact of this condition on their ­quality of life and mental health, underscoring the necessity for more individualized and empathetic therapeutic approaches^14^.

Conservative management includes self-management ­education, weight control and dietary advice, physiotherapy and exercise, compression therapy with a flat mesh, and ­manual lymphatic drainage, regularly. Patient education consists of informing them about the chronic and progressive condition and presenting all the treatment options. Considering that patients with lipedema have a high risk of developing obesity, weight control through dietary changes and exercise should be encouraged^15,16^. There is no dietary strategy for lipedema.

## THE SIGNIFICANCE OF EVIDENCE-BASED PRACTICE

Evidence-based practice is of paramount importance in the ­management of lipedema, as it allows the application of ­treatments based on robust scientific research. The lack of ­specific data on the prevalence and characteristics of lipedema in Brazil limits the effectiveness of interventions. Studies such as Melander's^17^ illustrate the experience of women with lipedema:

"*I found a picture of a woman who looked precisely like me, and she had lipedema. I decided to show the picture to my doctor. He, however, believed that this was just a way to put the blame on someone else. He said to me, ‘What are you doing here? Whose fault is it that you are fat? Is it your mother's, your children, or someone else? By the way, you have curves in the right place.’ I felt so humiliated and did not understand why he reacted like that*"^17^.

The first guidelines on lipedema, proposed in Germany in 2015, are an important step, but there is a continuous need to update and adapt them to different contexts. Two recent publications point the way toward therapeutic proposals for lipedema in terms of nutrition, the article "Ketogenic Diet as a Potential Intervention for Lipedema"^18^ and "Ketogenic Diet: A Nutritional Therapeutic Tool for Lipedema?"^19^.

They compete with a series of unsupervised low-carb and ketogenic diets and practices that are commonly disseminated in groups on the Internet but are also being prescribed by medical and nutritional professionals^6^. In a research setting, we are investigating the impact of such practices on women's body image and eating behavior. In a recent qualitative analysis, the patient reported during our interviews that she had heard from a Brazilian specialist doctor:

"*If you know it's bad for you, why eat it?*"

The reductionism of willpower and control in dietary management is already well documented in studies on the influence of weight stigma and fatphobia on health professionals and patients with lipedema. These patients seem to be at risk of developing eating disorders, mainly because of how diet culture interferes with the care and conduct of doctors and nutritionists. In one of their quantitative and qualitative studies, Fetzer and Fetzer^20^ collected reports from their patients with lipedema, which were similar to those of patients with eating disorders:

"*The psychological damage of this condition is as painful to deal with as the physical elements. Feeling alone and most people, including most doctors, assuming I'm lazy, greedy and fat.*"

"*It's depressing, it restricts my life. I just wish my body was less disgusting*"^20^.

Clinical practice must be based on evidence to offer ­effective and humane treatments. The therapeutic approach must be multidisciplinary, integrating medical, psychological, and nutritional care to address all facets of lipedema^21^. Data from the literature indicate the impact of stigma on the search for treatment and the emotional dysregulation and comorbid ­disorders present in these patients. Moreover, it is imperative that guidelines undergo continuous review and adaptation, incorporating new scientific findings and clinical experiences, to enhance patients’ quality of life^22-24^.
