# Erratum

**DOI:** 10.1590/1806-9282.20240801ERRATUM

**Published:** 2024-11-11

In the manuscript "Lipedema: clinical characteristics, complications, and the importance of evidence-based practice", https://doi.org/10.1590/1806-9282.20240801, published in the Rev Assoc Med Bras. 2024;70(9):e20240801 on page 1:

**Where it reads:**

**Table 1:** Presentations of lipedema.

Stages	Types
Stage I: The surface of the skin is normal, and the subcutaneous fatty tissue has a soft consistency. However, multiple small nodules can be palpated.	Type I: presents as an increase in fatty tissue on the buttocks and thighs.
Stage II: The surface of the skin becomes uneven and stiffer due to the increased formation of nodular structures (large lumps) in the subcutaneous fatty tissue, a process called liposclerosis.	Type II: involves the extension of edema and the formation of fatty deposits on the inside of the knees.

**It should read:**

**Table 1:** Presentations of lipedema.

Stages	Types
Stage I: The surface of the skin is normal, and the subcutaneous fatty tissue has a soft consistency. However, multiple small nodules can be palpated.	Type I: presents as an increase in fatty tissue on the buttocks and thighs.
Stage II: The surface of the skin becomes uneven and stiffer due to the increased formation of nodular structures (large lumps) in the subcutaneous fatty tissue, a process called liposclerosis.	Type II: involves the extension of edema and the formation of fatty deposits on the inside of the knees.
Stage III: This stage is characterized by lobular deformation of the skin's surface, resulting from an increase in adipose tissue. The nodules vary in size and can be clearly differentiated from the surrounding tissue on palpation. The phenomenon known as "peau d'orange" can be observed when pressing the skin.	Type III: has an extension from the hips to the ankles.
	Type IV: manifests itself in both the legs and arms.
	Type V: lipolymphedema.

